# ArrayQuest: a web resource for the analysis of DNA microarray data

**DOI:** 10.1186/1471-2105-6-287

**Published:** 2005-12-01

**Authors:** Gary L Argraves, Saurin Jani, Jeremy L Barth, W Scott Argraves

**Affiliations:** 1 Array Genetics, Inc., 59 Great Quarter Road, Newtown, CT 06482, USA; 2 Department of Cell Biology and Anatomy, Medical University of South Carolina, Charleston, SC 29425 USA

## Abstract

**Background:**

Numerous microarray analysis programs have been created through the efforts of Open Source software development projects. Providing browser-based interfaces that allow these programs to be executed over the Internet enhances the applicability and utility of these analytic software tools.

**Results:**

Here we present ArrayQuest, a web-based DNA microarray analysis process controller. Key features of ArrayQuest are that (1) it is capable of executing numerous analysis programs such as those written in R, BioPerl and C++; (2) new analysis programs can be added to ArrayQuest Methods Library at the request of users or developers; (3) input DNA microarray data can be selected from public databases (i.e., the Medical University of South Carolina (MUSC) DNA Microarray Database or Gene Expression Omnibus (GEO)) or it can be uploaded to the ArrayQuest center-point web server into a password-protected area; and (4) analysis jobs are distributed across computers configured in a backend cluster. To demonstrate the utility of ArrayQuest we have populated the methods library with methods for analysis of Affymetrix DNA microarray data.

**Conclusion:**

ArrayQuest enables browser-based implementation of DNA microarray data analysis programs that can be executed on a Linux-based platform. Importantly, ArrayQuest is a platform that will facilitate the distribution and implementation of new analysis algorithms and is therefore of use to both developers of analysis applications as well as users. ArrayQuest is freely available for use at .

## Background

Numerous Open Source projects contribute source code for routines that analyze DNA microarray data [1]. Projects such as Bioconductor [2] and Bioperl [3] offer web accessible repositories for DNA microarray software packages. However, obstacles such as lack of expertise in configuring the software for use on one's local workstation (i.e., installing operating systems such as R and packages that run under R) and inadequate hardware resources (i.e., computers with high CPU throughput) may prevent implementation of these tools by biologists. Providing browser-based interfaces that allow the programs to be executed over the Internet can enhance the applicability of these advanced analytic software tools by researchers. Here we describe a new web-based DNA microarray analysis process controller that permits application of Open Source programs to analyze DNA microarray data. ArrayQuest is designed as a broadly applicable process controller that will implement DNA microarray analysis programs executable on a Linux system. We believe that this feature can help biologists employ the wealth of Open Source software that has been developed for analyzing DNA microarray datasets.

There are a number of existing web-server applications that perform microarray data transformations such as data normalization, two-condition comparisons and unsupervised and supervised clustering [4-9]. In general these tools execute analysis steps sequentially, and the output from one analysis step is then input or specified prior to subsequent analysis. For example, tools such as *Expression Profiler: Next Generation *developed at EBI [10] will execute a normalization of hybridization data that is then output to a file. This data file must then be uploaded (perhaps after reformatting the data) before differentially expressed genes may be identified. Similarly, the differentially expressed genes must be output to a file and this data must be uploaded before any subsequent analysis can be conducted. We have designed ArrayQuest to execute analysis processes comprised of bundles of different routines, thereby simplifying the execution of multi-step analyses.

## Implementation

### ArrayQuest programming

ArrayQuest was written in PHP4 and Linux Bash Shell Scripting language and runs on multiple Linux RedHat servers. Project data, DNA microarray data supersets and analysis process control data are saved in a MySQL database. The backend analysis computers use Secure Shell (Shh) as the communication link to the center point web server. The system is designed to permit expanded analysis capacity by distributing analysis requests to servers outside of the local area network.

### ArrayQuest usage

**Figure 1:**
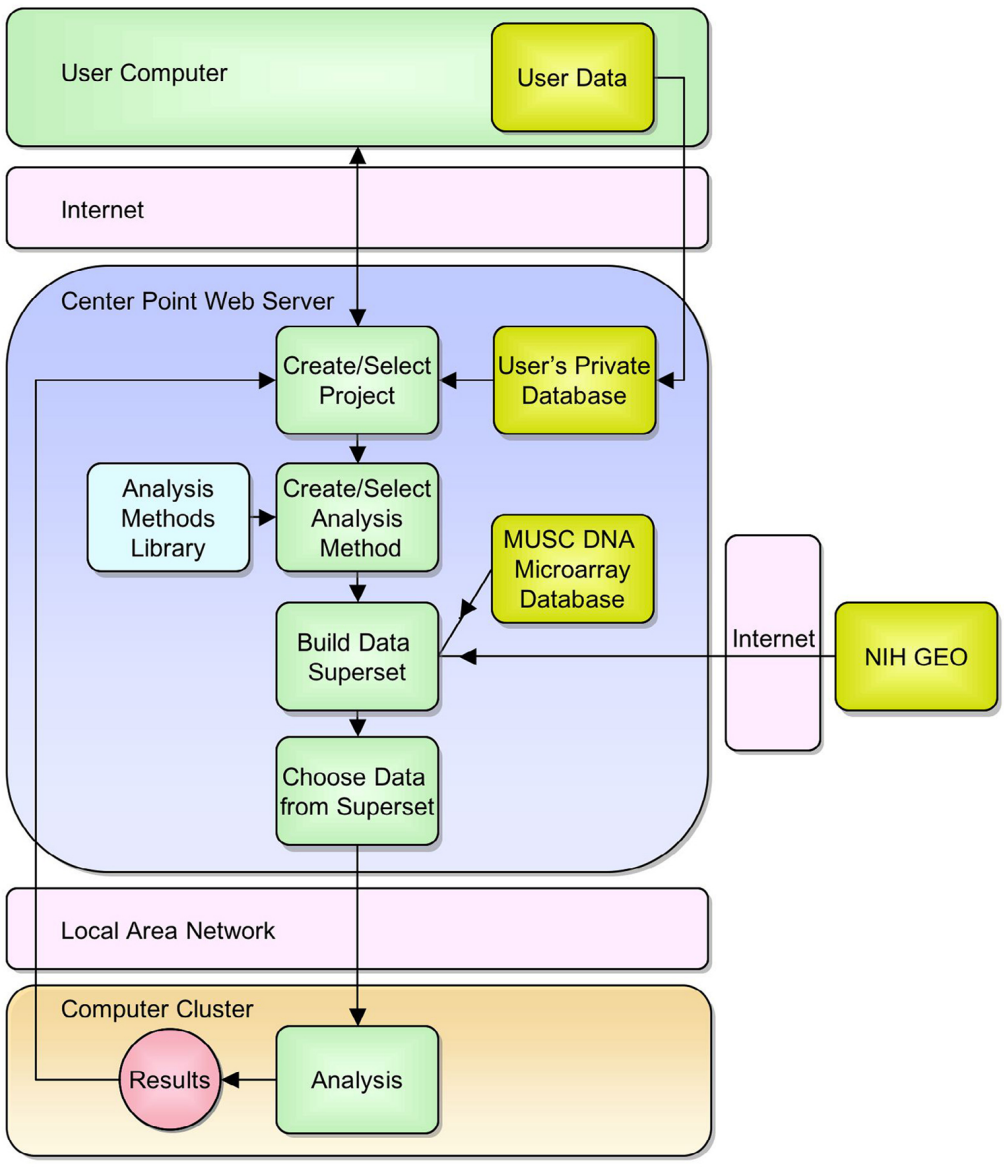
**Schematic diagram depicting ArrayQuest system topography and steps in the process of performing an analysis of DNA microarray data**. As indicated, DNA microarray data can be obtained from multiple sources including the MUSC DNA Microarray Database, the NIH GEO database or a user's private database.

ArrayQuest [11] is accessible through web browsing portals (i.e., Internet Explorer, Safari, Mozilla, etc.) and can be used after registering to obtain a free password-protected account. The ArrayQuest system allows Internet clients to connect to a center point web-server to create a new analysis project or select an existing project folder (Fig. 1). Project folders contain Principal Investigator (PI) and project information entered via a web form. Once a project folder has been created, the user may choose to upload data files from their computer to create their own private database. Next, a single analysis method is selected from methods that are stored in the Methods Library. Depending on the method chosen, input microarray data for an analysis can be selected from either the MUSC DNA Microarray Database [12], a remote database (e.g., GEO) or the user's private database containing data uploaded to the center point web-server. Upon launching an analysis process, the data required to perform the analysis is sent to a computer belonging to a cluster of analysis computers. At the Medical University of South Carolina, the hardware configuration for a computer within the ArrayQuest analysis computer cluster is a dual Opteron CPU (AMD 64 bit) having 2 gigabyte of RAM, running under Fedora Core. Each computer in the cluster is loaded with the R programming language [13] and Bioconductor software packages [2,14]. Besides R and Bioconductor analysis tools, the system is capable of executing other types of analysis programs (e.g., BioPerl and C++).

### Results reporting

ArrayQuest automatically recognizes result files that are produced by an analysis script based on file time tagging. When an analysis script has completed execution, the files are copied from the analysis computer back to the center point web server for viewing (Fig. 1). When requests require lengthy run times, the system will notify the user upon completion by email. The script status can also be queried in real time through the web browser to determine whether an analysis job is running or completed, the duration of the analysis, whether an error has occurred and which analysis computer in the cluster is running the analysis process.

### User management and privileges

The system has two access levels, administrator and user. All users are allowed to work in a password-protected environment, private from all other users. Users have access to all publicly available data in the MUSC DNA Microarray Database as well as any privately held data that they have deposited in the database. Users cannot create or modify analysis methods but can work with administrators to have methods developed and/or implemented. Lists of users will never be shared with a third party. The system will also send (email) forgotten passwords to users on request.

### Adding new analysis methods to ArrayQuest

New analysis methods can be added to the Methods Library at the request of users or developers. A method developer may submit a program description or script to the ArrayQuest system administrator for consideration [11]. Once approved, ArrayQuest personnel will aide in the implementation of the method on the ArrayQuest platform. Non-developers may also request that ArrayQuest personnel create a method from existing Open Source programs. Since the specification of analysis parameters can be conducted entirely via a parameters entry text window, new methods do not require that graphical user interfaces (GUIs) be created. This alleviates some of the burden of implementing new analysis methods that may be encountered with other online analysis tools that use GUIs for setting analysis parameters.

## Results and discussion

There are a number of existing web-server applications that perform common data transformations such as data pre-processing, two-condition comparisons and unsupervised and supervised clustering [4-9]. One feature that distinguishes ArrayQuest from these other applications is that ArrayQuest is designed as a broadly applicable process controller that will implement DNA microarray analysis programs executable on a Linux system. Therefore, ArrayQuest is not limited to execution of a subset of specific analysis functions, but is instead capable of executing a large number of analysis functions that have been generated by the Open Source community and that can be implemented in a Linux-based system.

The ArrayQuest Methods Library is currently populated with six methods for analysis of Affymetrix DNA microarray data. These include a number of Bioconductor-based statistical and graphical methods written in R that accept Affymetrix .CEL files and one method that accepts GEO GDS .SOFT files (Table 1). The methods range in complexity from simple data normalization (method 10) to the more comprehensive procedure of normalization, identification of differentially expressed genes, hierarchical clustering, generation of a heatmap and significance analysis of gene ontologies (method 12). In general, individual methods were created by combining or "stacking" Bioconductor packages in order to execute a series of linked analysis routines. Depending on the method selected, users will be required to specify input parameters for parsing of the data such as control and experimental filenames, thresholds for differential expression (e.g., fold change, t-test *p*-value and false discovery rate) and/or GO IDs.

**Table 1:** Representative analysis methods held in the ArrayQuest Methods Library.

Method Title	Method Description	Required Data Format	Data Source	Programming Language/ Software^1^	Output
RMA Normalization of Affymetrix Data	This method performs Robust Multichip Analysis (RMA) to generate normalized expression intensities for a set of Affymetrix GeneChip CEL files.	Affymetrix GeneChip data in .CEL file format	MUSC DNA Microarray Database or User's Private Database	R/Bioconductor	A Microsoft Excel file of normalized intensities transformed into log base 2 for all genes and four JPEG files of box plots and histograms of expression intensities before and after normalization.
Identification of differentially expressed genes based on fold-change, p-value and/or FDR parameters	This method is used to analyze data from any two-condition microarray experiment. The algorithm normalizes hybridization data, finds differentially expressed genes based on fold-change, t-test and FDR thresholds, collects annotations for these genes, performs hierarchical clustering and renders a heat map of the expression profiles.	Affymetrix GeneChip data in .CEL file format	MUSC DNA Microarray Database or User's Private Database	R/Bioconductor	Annotation reports (Excel and HTML); Heatmap of differentially expressed genes (.JPEG); KEGG pathway heat maps of differentially expressed genes (as many as are found) (.JPEG); GO Information (HTML).
Identification of differentially expressed genes based on p-value, fold-change and/or FDR parameters: .SOFT files only	This method is used to analyze Affymetrix DNA microarray data that can be obtained from NIH GEO as a .SOFT.gz file. The method normalizes hybridization data (RMA), finds differentially expressed genes based on fold-change, t-test and FDR thresholds, collects annotations for these genes, performs hierarchical clustering and renders a heat map of the expression profiles.	Affymetrix GeneChip data in GEO .SOFT file format	Gene Expression Omnibus (GEO)	R/Bioconductor	Annotation reports (Excel and HTML); Heatmap of differentially expressed genes (.JPEG); KEGG pathway heat maps of differentially expressed genes (as many as are found) (.JPEG); GO Information (HTML).
Assessment of gene expression associated with a specified GO ID(s)	This method analyzes Affymetrix GeneChip data to find gene expression values associated with specified GO IDs. The script normalizes GeneChip hybridization data (RMA), extracts hybridization values for genes associated with a user-provided GO ID, performs hierarchical clustering and renders a heat map of the expression profiles.	Affymetrix GeneChip data in .CEL file format	MUSC DNA Microarray Database or User's Private Database	R/Bioconductor	Boxplots and histograms of expression intensities before and after normalization (each in JPEG). Heat map based on the number of GO IDs provided by the user (each in JPEG).

^1^ArrayQuest methods displayed in this table are written using the R statistical computing language [13] and implement packages/algorithms that are the product of the Bioconductor Open Source software development project [2]. Bioconductor packages used in ArrayQuest methods and links to package descriptions and developers are given in the description window for each method in the ArrayQuest Methods Library [11].

The ability to execute bundled analyses in one step is another feature that distinguishes ArrayQuest from other online microarray analysis tools. For example, after specifying a group of raw hybridization data files Method 12 will identify differentially expressed genes, find significantly represented gene ontologies and perform hierarchical clustering. Online tools that perform each of these steps independently require that the user continuously interface with the website to enact each step. This tends to increase the time required to execute the analysis and can increase the complexity and difficulty of the analysis. By combining all stages of the analysis into a single process, as is the ability of ArrayQuest, the overall analysis process is significantly simplified and speeded up. A typical execution of Method 12 involving two sample groups (≤ 5 replicates each) may be completed in approximately 10 minutes.

## Conclusion

ArrayQuest will serve as a useful system for analysis of DNA microarray data on-line and will also enable software developers to make their DNA microarray analysis routines readily available to the research community.

## Availability and requirements

Project name: ArrayQuest

Project home page: 

Operating system(s): Operates online via a browser web portal. Web servers use Redhat Linux and Fedora Linux.

Programming language: PHP4 patched to support file uploading status ); bash and standard Linux system utilities.

Other requirements: Apache (HTTP server), mySQL (Structured Query Language server), ssh (Secure Shell), awk, scp (secure copy) and microArrayDB (μArrayDB; ).

License: GNU General Public License (GPL) and BSD as applicable to subsystems of ArrayQuest.

Any restrictions to use by non-academics: Not at this time.

Anonymous review of ArrayQuest: ArrayQuest can be accessed in an anonymous fashion at  using the guest user account (Username / password: test@yahoo.com / test). This account is populated with a project containing two analyses. One of these (Sample Analysis Process I) is intended as an analysis that the user can modify, execute and then check analysis results. The other (Sample Analysis Process II) should not be modified and is intended only as an example of the analysis procedure and a demonstration of analysis output.

Analysis script availability: Analysis scripts employed in ArrayQuest methods are freely available and can be found on the Methods Library List page () by toggling the "Script" button associated with every analysis method.

## List of abbreviations used

GEO, Gene Expression Omnibus; GO ID, Gene Ontology ID; MUSC, Medical University of South Carolina.

## Authors' contributions

Gary L. Argraves is the primary designer and systems programmer of ArrayQuest.

Saurin Jani worked to implement R-based Bioconductor routines to run on ArrayQuest and consulted on system features and debugging.

Jeremy L. Barth directed the creation of ArrayQuest analysis process routines.

W. Scott Argraves is a designer of ArrayQuest and Principal Investigator on the NIH grant that funded the project.
